# Architecture and Expression of the *Nfatc1* Gene in Lymphocytes

**DOI:** 10.3389/fimmu.2014.00021

**Published:** 2014-02-03

**Authors:** Ronald Rudolf, Rhoda Busch, Amiya K. Patra, Khalid Muhammad, Andris Avots, Jean-Christophe Andrau, Stefan Klein-Hessling, Edgar Serfling

**Affiliations:** ^1^Department of Molecular Pathology, Comprehensive Cancer Center Mainfranken, Institute of Pathology, University of Würzburg, Würzburg, Germany; ^2^Centre d’Immunologie de Marseille-Luminy, Universite Aix-Marseille, Marseille, France

**Keywords:** chromatin, induction, lymphocytes, *Nfatc1*, transcription

## Abstract

In lymphocytes, the three NFAT factors NFATc1 (also designated as NFAT2), NFATc2 (NFAT1), and NFATc3 (NFAT4 or NFATx) are expressed and are the targets of immune receptor signals, which lead to a rapid rise of intracellular Ca^++^, the activation of phosphatase calcineurin, and to the activation of cytosolic NFATc proteins. In addition to rapid activation of NFAT factors, immune receptor signals lead to accumulation of the short NFATc1/αA isoform in lymphocytes which controls their proliferation and survival. In this mini-review, we summarize our current knowledge on the structure and transcription of the *Nfatc1* gene in lymphocytes, which is controlled by two promoters, two poly A addition sites and a remote downstream enhancer. The *Nfatc1* gene resembles numerous primary response genes (PRGs) induced by LPS in macrophages. Similar to the PRG promoters, the *Nfatc1* promoter region is organized in CpG islands, forms DNase I hypersensitive sites, and is marked by histone tail modifications before induction. By studying gene induction in lymphocytes in detail, it will be important to elucidate whether the properties of the *Nfatc1* induction are not only typical for the *Nfatc1* gene but also for other transcription factor genes expressed in lymphocytes.

## Introduction

In peripheral B lymphocytes, the NFATc factors NFATc1, c2, and c3 are the final targets of B cell receptor (BCR)-mediated activation, and inhibiting their induction by the immunosuppressant Cyclosporin A (CsA) abrogates the antigen-induced proliferation of B cells (1). In freshly isolated (naive) splenic B cells according to the number of RNA reads in RNA Seq assays, 10-fold more transcripts were detected for the *Nfatc1* and *Nfatc3* genes than for the *Nfatc2* gene. BCR signals increase the transcription of the *Nfatc1* gene, but not of the *Nfatc2* and *Nfatc3* genes (Muhammad et al., submitted). Although all three NFATc transcription factors (TFs) bind to similar DNA motifs and transactivate the promoters of numerous genes in transfection studies, inactivating the individual *Nfatc* genes in mice resulted in quite diverging phenotypes. Whereas inactivation of the *Nfatc1* gene led to an early death of mice embryos (2, 3), *Nfatc2^−/−^* mice were born at normal Mendelian ratio but developed with age, a hyper-proliferative syndrome and elevated immune responses (4–6). These features of the *Nfatc2^−/−^* mice were found to be accelerated in mice deficient in both NFATc2 and NFATc3 (7). Ablation of NFATc1 in B cells led to a marked reduction in BCR-mediated proliferation and Ca^++^ flux, increase in activation induced cell death (AICD), and defects in antibody production upon immunization, whereas opposite effects were observed for *Nfatc2^−/−^* B cells (1, 8).

These functional differences between NFATc1 and NFATc2 might be due to the synthesis of NFATc1/αA, a short isoform of NFATc1, which lacks the C-terminal peptide of approximately 250 amino acids residues typical for most of the other NFATc proteins. NFATc1/αA is the most prominent NFAT protein in effector B cells and is able to rescue B cells from early cell death (1).

## Structure of the *Nfatc1* Gene

**Figure 1:**
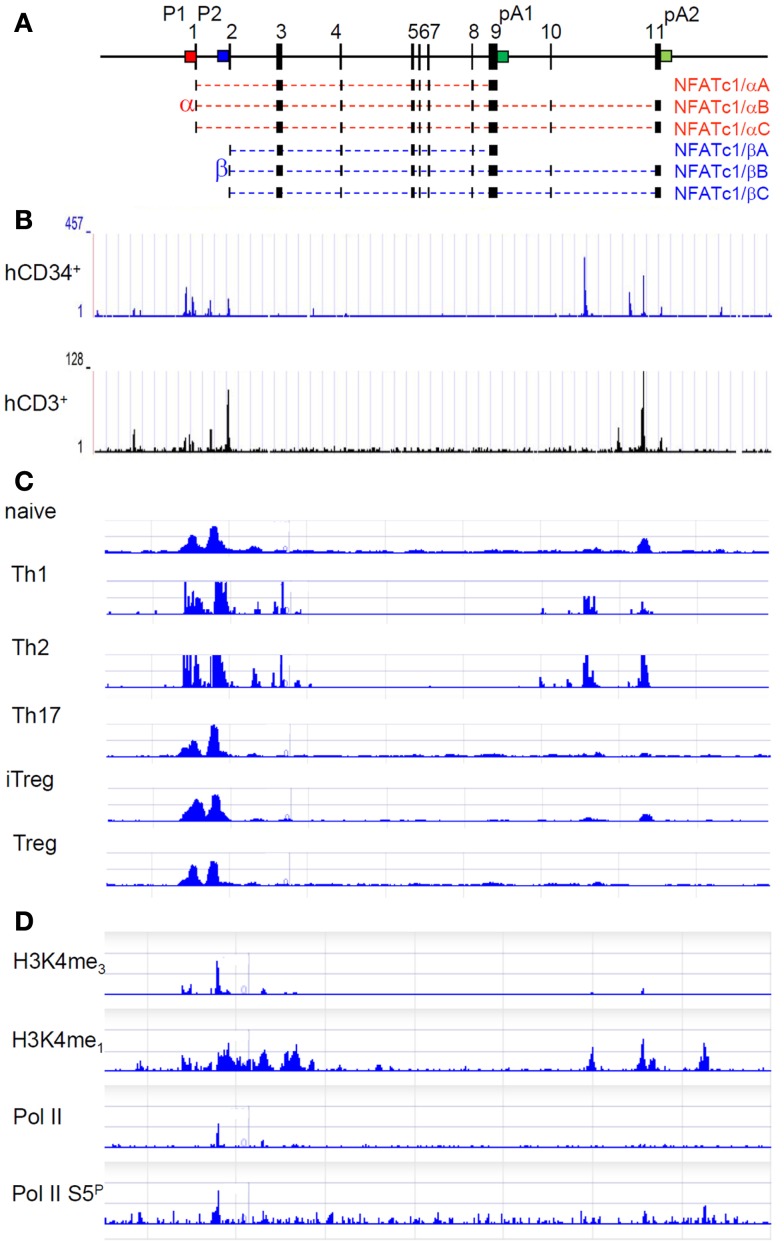
**Molecular organization and epigenetic marks of the *Nfatc1* gene**. **(A)** Exon–intron structure of the murine *Nfatc1* gene. The two promoters P1 (red) and P2 (blue) and the two poly A addition sites pA1 and pA2 (both in green) are indicated. In intron 10, an enhancer for the *Nfatc1* induction in lymphocytes is located, and at the 3′ end of intron 1, an enhancer was described for controlling *Nfatc1* transcription in endocardial cells of the developing heart (13). **(B)** Occurrence of DNase I hypersensitive sites in the human *NFATC1* gene in CD34^+^ hematopoietic progenitor cells and CD3^+^ T cells (see http://nihroadmap.nih.gov/epigenomics (14). Note the existence of DNase I hypersensitivity over the promoter region and the intron 10 in which we identified an enhancer for the induction of *Nfatc1* gene (Patra et al., in preparation; see below). **(C)** Epigenetic H3K4me3 marks – as indicator for regulatory elements, which are either poised for or active in transcription (15) – at the promoter region and intron 10 sites in Th1 and Th2 cells (16). Note the appearance of H3K4me3 mark around the P1 and P2 promoters in all types of T cells, whereas the occurrence of H3K4me3 mark in intron 10 is restricted to Th1 and Th2 in which we observed a strong *Nfatc1* induction upon cellular activation (17). **(D)** Accumulation of RNA polymerase II (pol II) and of epigenetic marks on the *Nfatc1* gene in DP thymocytes (18). Note the appearance of pol II at promoter P2 and of enhancer mark H3K4me1 in the promoter region and intron 10.

The genes encoding NFATc1 in mouse and man consist of 11 exons and span approximately 110 and 134 kb DNA, respectively. Due to the existence of two promoters, two poly A sites and alternate splicing events, six NFATc1 RNAs, and proteins are generated in peripheral lymphocytes (9–11) (Figure 1A). The two *Nfatc1* promoters, P1 and P2, show the typical features of eukaryotic promoters. They are highly conserved between mouse and man over 800 bp (P1) or 100 bp (P2) DNA and form DNase I hypersensitive chromatin sites. Both promoters are organized in CpG islands. While in peripheral blood lymphocytes, in Jurkat T cells, and in other lymphoid cell lines in which NFATc1 is expressed the DNA of promoter islands is de-methylated, inactivation of human *NFATC1* gene in several Hodgkin’s lymphoma cells lines is correlated with the methylation of all CpG dinucleotides within the P1 promoter (12).

The inducible P1 promoter of 800 bp can be divided into two DNA “homology blocks” (10) of approximately 250 bp DNA, which harbor DNA binding motifs for Sp1 at their termini. Sp1 binding is known to protect CpG islands from DNA methylation (19, 20), and the relatively weak binding of Sp1 to these P1 sites in Hodgkin’s lymphoma cells in which the promoter is suppressed led us to speculate that they could function as “road blocks” to prevent the methylation of P1 DNA in effector lymphocytes (12) [but see also Ref. (21) and below for the function of Sp1 in the control of primary response promoters]. The TF binding motifs within each block of homology represent composite sites for the inducible binding of Creb/Fos/ATF and NF-κB/NFAT factors. When used as probes in electrophoretic band shift assays (EMSAs), the NF-κB/NFAT sites form predominantly NF-κB complexes with proteins from activated T and B cells. However, under conditions of high nuclear NFAT levels these sites can also be bound by NFAT. Together with the two NFAT sites within the distal block of homology, which are arranged in tandem, they enable strong binding of NFAT factors to the P1 promoter (9). This contributes to the NFATc1-mediated auto-regulation of P1-directed *Nfatc1* transcription, which keeps high levels of NFATc1 for days under persistent immune receptor stimulation (11).

In spite of the tight assembly of TF binding sites within the P1 promoter, when transfected into EL-4 thymoma cells, P1-directed luciferase reporter constructs showed a poor induction, which differs markedly from the induction of endogenous *Nfatc1* gene (9). Instead of being induced as the endogenous *Nfatc1* gene by the phorbol ester TPA and the Ca^++^-ionophore ionomycin (which mimic immune receptor signals), inducers of protein kinase A, such as forskolin, led in combination with ionomycin to the strongest induction of P1 in EL-4 thymoma cells (9).

These functional studies on the induction of *Nfatc1* P1 promoter led us to conclude that not all sequence elements controlling the induction of the *Nfatc1* gene in lymphocytes are part of its promoter region. Whereas fusion of more upstream DNA to the highly conserved P1 region of approximately 800 bp did not result in any increase in promoter induction, fusion of a 1-kb DNA fragment from the central region of intron 1 of the *Nfatc1* gene to P1 enhanced its overall activity fourfold to fivefold, but did not affect its induction mode (9). However, when we inserted an element from intron 10 of the *Nfatc1* gene into a luciferase construct directed by the P1 (or P2) promoter, we observed both a strong increase in promoter induction and a mode of induction similar to the endogenous *Nfatc1* gene. These findings suggest that an enhancer for the optimal induction of *Nfatc1* gene is located in intron 10, which supports *Nfatc1* induction in lymphocytes (Patra et al., in preparation).

In Figures 1B,C, mapping studies of DNase I hypersensitive sites in human CD34^+^ lymphoid progenitor cells and CD3^+^ T cells and of H3K4me3 mark in various subsets of murine T cells are presented for the *Nfatc1* gene. They show that, in addition to the promoter region, both DNase I hypersensitive chromatin sites and H3K4me3 marks were mapped within intron 10 of *Nfatc1*. The distal intron 10 site was found to be marked by H3K4me1 and H3K4me3 modifications (see Figures 1C,D), and the enrichment of H3K4me3 was identified as a feature of active enhancers in T cells (22). And indeed, we determined this site (designated as E2) as an enhancer element that supports the induction of P1 and P2 promoters in lymphocytes (Patra et al., in preparation). Interestingly, E2 appears to be less active (or inactive) in Th17 cells, in thymus-derived regulatory T cells (Treg), and in induced Treg (iTreg) in which we observed a weak *Nfatc1* induction (17).

In resting CD4^+^ T cells and DP thymocytes in which *Nfatc1* is poorly expressed, the (P2) promoter region of the *Nfatc1* gene shows characteristics of a “transcription initiation platform.” In ChIP-Seq assays using DP thymocytes, the RNA polymerase II (pol II) was found to be bound at P2 (and not at P1), whereas the enhancer mark H3K4me1 was detected over the entire promoter region and several intron 10 sites (Figure 1D) (18). However, when ChIP assays were performed for H3K27ac, a mark for active – and not only poised – enhancers (23), only a peak over the central intron 10 enhancer segment E2 appeared in double-negative (DN) thymocytes (Andrau, in preparation) in which the *Nfatc1* gene is expressed more robustly than in DP thymocytes (17).

## *Nfatc1* Expression in Peripheral B Cells

When splenic B cells are induced by α-IgM for 24 h *ex vivo*, the predominant synthesis of short NFATc1 isoform NFATc1/αA is observed (1, 17). While in Western blots using whole B cell protein a strong, more than 50-fold induction of NFATc1/αA protein is detected, in real time PCR assays measuring the levels of *Nfatc1/*α*A* RNA a 5- to 10-fold increase was observed, and in recent RNA Seq assays, high levels of NFATc1 RNA were found in non-stimulated primary splenic B cells, which are not reflected at the protein level. These observations suggest the existence of both transcriptional and post-transcriptional control mechanisms, which shape the appearance of NFATc1 protein(s) upon B (and T) cell induction.

To study the expression of *Nfatc1* gene at the transcriptional level *in vivo*, we generated a BAC transgenic (tg) mouse line, which expresses an *Egfp* reporter gene under the control of the entire *Nfatc1* locus (17). Within the BAC construct, the *Egfp* reporter replaces exon 3 of the *Nfatc1* gene followed by a SV40 poly A addition signal, which gives rise to short chimeric *Nfatc1/Egfp* RNAs and proteins. Therefore, the *Nfatc1/Egfp* transcripts are generated under the control of all regulatory elements of the *Nfatc1* gene, including both promoters and the downstream enhancer. However, the post-transcriptional mechanisms leading to NFATc1/αA protein differ certainly between “normal” *Nfatc1* and *Nfatc1/Egfp* transcripts. Thus, in lymphocytes of tg *Nfatc1/Egfp* mice, the expression of chimeric *Nfatc1/Egfp* tg should reflect the transcription of *Nfatc1* locus, but not the expression of NFATc1 proteins.

In tg *Nfatc1/Egfp* mice, the *Nfatc1* gene is expressed as early as in DN thymocytes and in naïve resting T and B cells of peripheral lymphoid organs. Although before the induction of pre-T cell receptor at the transition of DN3 to DN4 thymocytes, NFATc1 α-isoforms are not generated and, therefore, the P1 promoter is less active (or inactive), the *Nfatc1* gene appears to be transcribed at a relatively high level in DN thymocytes lacking any immune receptor (Patra et al., in preparation). This appears also to be the case in naïve and resting T and B lymphocytes. Thus, similar to other TF genes encoding Fos, Jun, Egr, ATF, and further TF factors, which harbor CpG islands in their promoters, the *Nfatc1* gene seems to belong to the group of primary response genes (PRGs) that show a moderate 5- to 10-fold induction upon cellular stimulation. Contrary to secondary response genes (SRGs), which are often induced more than 100-fold, PRGs appear to be organized in an “open” chromatin, which is poised for transcription or transcribed at a low level (24, 25).

To a large part, our current view on the regulation of inducible genes bases on studies about LPS-mediated gene induction in macrophages (21, 26), see also (27) and (24). Previous approaches on the knock down of components of SWI/SNF nucleosome remodeling complexes in macrophages showed that in contrast to the SWI/SNF-dependent induction of SRGs, the LPS-mediated induction of PRGs is independent of SWI/SNF (28). Similar to promoters of many house-keeping genes, which are also organized in CpG islands, PRG promoters exhibit constitutively active chromatin with unstable nucleosomes, which form constitutive DNase I hypersensitive regions (26). Before induction, they are associated with the initiating version of pol II phosphorylated at “Ser5” within their C-terminal domain (CTD), and with Sp1, which helps to recruit pol II. But contrary to the heat shock genes in *Drosophila*, which are also pre-loaded with pol II and transcribed into short RNAs (29), PRG transcripts in macrophages are elongated to full-length transcripts, which appear to be instable and un-spliced (21). LPS stimulation, however, which often leads to binding of NF-κB to the promoters of PRGs results in the phosphorylation of pol II at position S2 within its CTD repeats and the generation of stable RNAs, which are spliced and processed (21, 26).

The architecture of the *Nfatc1* promoter region and its induction is similar, but not in all aspects, to PRG promoters and their induction in macrophages. In lymphocytes, induction of the *Nfatc1* gene is controlled predominantly by immune receptor signals but not by LPS [or other co-stimulatory signals; see Ref. (1)]. The *Nfatc1* promoter region is organized in CpG islands, forms DNase I hypersensitive sites, and is bound by Sp1 [and CREB, which controls activity-dependent PRG regulation in neurons (30)] prior to its induction by NF-κB. However, induction of the *Nfatc1* promoter differs significantly from that of PRG promoters in macrophages. In contrast to PRGs (31) and similar to the “inducible house-keeping” Nfkbia gene (21), the *Nfatc1* gene is efficiently transcribed in lymphocytes prior to the appearance of stable, spliced transcripts in response to receptor signals. These transcripts, however, remain un-translated (Muhammad et al., in preparation.).

## Summary and Implications

The immune receptor-mediated induction of NFATc1 TFs in peripheral lymphocytes can be divided in two events: (i) the rapid nuclear transport and activation of pre-formed cytosolic NFATc proteins, and (ii) the massive transcriptional and post-transcriptional induction of NFATc1/αA, a short NFATc1 protein, which differs in many properties from other NFATc proteins (10). Although the induction of the *Nfatc1* gene leading to NFATc1/αA in lymphocytes resembles the LPS-mediated induction of PRGs in macrophages, it appears to differ from the induction of many PRGs by (i) its high constitutive transcription into spliced transcripts and (ii) its enhancer-mediated control. While the molecular details of these events remain to be elucidated, it will be important to investigate whether the properties of *Nfatc1* induction are specific for the *Nfatc1* gene or a property of immune receptor-mediated induction of many TF genes in lymphocytes. In any way, the detailed knowledge of molecular mechanisms controlling the induction of NFATc1 in lymphocytes could pave the way to interfere with its induction, which controls numerous aspects of adaptive immunity.

## Conflict of Interest Statement

The authors declare that the research was conducted in the absence of any commercial or financial relationships that could be construed as a potential conflict of interest.
